# Situated Psychology: A Sketch

**DOI:** 10.1007/s12124-025-09927-2

**Published:** 2025-07-31

**Authors:** Rasmus Birk, Sarah Kirkegaard Jensen, Noomi Matthiesen, Bo Allesøe Christensen

**Affiliations:** Department of Communication and Psychology, Aalborg University, Aalborg, Denmark

**Keywords:** William james, Jean lave, Situated learning, Externalism

## Abstract

This article—and this special issue—focuses on situated psychology. The purpose of this article is to provide a brief theoretical outline of what situated psychology is. If the reader wonders why they are not immediately familiar with the term ‘situated psychology,’ there is a simple explanation: it is a new concept (except for Phillip Cushman’s argument that psychology should be “historically situated” (Cushman, 1990). However, despite the novelty of the name for the concept, it draws on a wide range of psychological traditions—ecological psychology, situated learning theory, 4E cognition, critical psychology, and more. The ambition of this article is thus to unfold a multifaceted theoretical perspective that understands situatedness as involving environment/culture/relations/context, but also as something other than just a framework within which human behavior unfolds.

The purpose of this article is to provide a theoretical outline of the notion of *situated psychology* which is the topic of this special issue.

If the reader wonders why they are not immediately familiar with the term ‘situated psychology,’ there is a simple explanation: the term *situated psychology* does not exist in the psychological literature as such.

(with the exception of Phillip Cushman’s argument that psychology should be “historically situated” (Cushman, 1990). However, despite the novelty of the name, what we will show in this paper is that there is a long tradition of thinking about psychological phenomena in a situated, contextualized, externalist (etc.!) fashion. From the beginning of psychology as a scientific discipline there has been a rich tradition for thinking about how psychological phenomena are, in our parlance here, *situated in the world*. Such traditions include, but are not limited to, ecological psychology, situated learning theory, 4E cognition and distributed cognition (e.g. Osbeck & Nersessian, 2013), critical psychology (e.g. Schraube, 2013), functionalism (e.g. Angell, 1907), cultural psychology and many other traditions of psychological thought. In this paper, as we argue that psychology – which includes both psychological phenomena and the psychological discipline - is situated, we thus draw attention to a number of common features in a broad, diverse, and (in some places) somewhat marginalized tradition in psychology that dates back to the early beginnings of scientific psychology. Here, the psyche and the psychological have been theorized as dependent on historical, material, social, relational, and/or cultural realities. Broadly, ideas range from suggesting that the psychological unfolds *in relation* to our environment and relationships to the more radical claim (e.g. in externalist perspectives) that the psychological *resides in* the world rather than ‘in the head’. Later in the article we devote some time to explicating the ideas of William James, but other early psychologists deserve a brief mention. Take, for example, James Rowland Angell, whose paper on *functional psychology* from 1907 functionalism up against the structuralist psychology of the late 1800s (as e.g. popularized by Edward Titchener) (Angell, 1907). For Angell, the province of functional psychology was one which focused on “the typical *operations* of consciousness under actual life conditions […]” (Angell, 1907, p.62–63, emphasis in original). For Angell, referencing Aristotle explicitly, “functional psychology” argued (in part) that any mental state, any experience of consciousness, is “dependent upon the particular exigencies and conditions which call them forth […] the affective coloring of such a psychical moment depend[s] upon one’s temporary condition, mood and aims […]” (Angell, 1907, p.67). Or, as he put it later, functionalism “[…] deals with the problem of mind conceived as primarily engaged in mediating between the environment and the needs of the organism.” (Angell, 1907, p.85). The psychological, in the sense given by Angell here (and which we shall also see later from William James) thus mediates between organism and world. To put the point slightly differently, even in these early ideas from functionalism, there was a sense that that *mind* was situated in and responsive to the world. As noted by Osbeck and Nersessian (2013), there are many similarities (and differences) between functional psychology and later approaches including what is sometimes called distributed cognition or 4E cognition, a tradition which we discuss in depth later in the paper. Another and – admittedly broader – example of how the situatedness of psychological phenomena has been a recurring topic is the early 20th century school of *Gestalt* psychology^1^. The gestalt psychologists emphasized the *context* of perception, for example in their famous visual illusions that demonstrate the relationship between the figure and its ground. Perception, in this sense, is situated in the world: to hear a song is not just to perceive individual sounds as they impinge upon the eardrums (Rock & Palmer, 1990), it is to perceive the *whole*, and the context of this perception matters as well. Broadly, then, the Gestalt psychologists can be said to have been interested in how psychological phenomena – in this case perception – were contextualized by the relation between the mind and the world. As these brief historical examples should demonstrate, the history of psychology, as it has crystallized in a multitude of both intertwined and diverse theoretical systems and practices, revolves around the question of the situated, albeit often under different terms, such as ‘environment’ (see e.g. Costall, 2004; Palmer, 2004). In addition to the above examples, across psychology—and its related disciplines—we find countless discussions about whether and in what ways people’s surroundings, environments, upbringing, context, and much more have influenced their personality, actions, inner experiences, behavior, and subject positions. This paper, then, draws (somewhat eclectically) on this rich vein of thought in psychology, focusing on a fundamental and shared insight, namely that *the psychological* does not reside purely ‘inside the head’. Our ambition is to unfold a multifaceted and *open-ended* theoretical perspective that understands environment, culture, relations, context (etc) as something other than a framework within which human behavior unfolds. What happens, in other words, if we theorize psychological phenomena as genuinely *situated in* the world? Situated psychology, in this sense, is meant as an open-ended and sensitizing concept (Blumer, 1954), meant to draw our attention to other ways of thinking and theorizing the psychological.

We proceed by reviewing and discussing four different theoretical understandings of the situated, which we will argue pave the way for the development of a distinct situated psychology. We want to be clear that there are other traditions that we do not discuss (even if they touch on similar ideas), such as critical psychology (Schraube, 2013). We begin the article with William James (1890/1950), whose philosophical and psychological ideas emphasized that psychology as a science should begin with and focus on the mental as it unfolds in concrete relationships. James provides one – but of course not the only – early example of how psychological phenomena could be understood in a situated way. The next perspective comes from anthropology, as we include Jean Lave’s work on situated learning (Lave, 2011), which understands human becoming as embedded in social practice. The third perspective is psychological externalism, a tradition wherein contemporary philosophers and psychologists have, in recent decades, discussed whether the mental can also be understood as being outside the body. A contemporary development of this, we will claim, is the tradition known as 4E cognition, which we review and discuss, focusing on how this perspective situates people’s psychological capacities and dispositions (Brinkmann, 2009) in relation to their surrounding environment. It is also a tradition that has been in dialogue with the ecological approach to psychology (cf. Clark, 1999; McGann et al., 2020), as formulated by, for example, James Gibson (J. J. Gibson, 1979/2015) and his successors. Our fourth and final perspective develops a historical approach to the situated nature of psychological phenomena. The perspectives that we explore in the article share an emphasis on the situatedness of the psychological; that psychological phenomena are situated in and gain their meaning from particular contexts and situations. For that very reason, a historical perspective is crucial. Situated psychology, in the sense that we give it here, implies an understanding of the historical, precisely to avoid ending up in a kind of situationism that may explain the immediate but not necessarily capture the historical contexts that make concrete situations possible and meaningful. To do this, we draw on, among others, the historian of psychology Kurt Danziger. The article concludes by formulating a proposal of what connects these four approaches in order to formulate key aspects of what could be termed a situated psychology.

## The Conditions of Mental Life

As mentioned, we begin this article with the famous American psychologist (and philosopher) William James (1842–1910). Of course, as we have discussed earlier in the paper, one could also have taken other historical starting points: to give another example, John Dewey also emphasized that experience depends on the situation (Brinkmann, 2011, p. 306; Dewey, 1896). Dewey and James, of course, knew of each other’s work, and both were deeply inspired by Charles Darwin’s ideas about evolution, which, in a way, also focused on the organism’s concrete situation in an environment. Our use of James in what follows should thus be understood as an example of how some of the ideas of situated psychology have roots in the early thinkers of the discipline, not as if James was the first or only early thinker to emphasize the concrete and situated character of the psychological.

James is, nowadays, perhaps most famous as the author of one of the first comprehensive works on psychology, namely *The Principles of Psychology*. *Principles* was originally published in 1890, and although it was a landmark volume at its time, influencing many early psychologists, it is rarely read today or considered anything more than a historical reference point for the origins of psychology as a discipline. But as we will briefly show in this section, both *Principles* and James’ later works are a useful starting point for developing a contemporary situated psychology.

The first two sentences in *The Principles of Psychology* are as follows: “Psychology is the Science of Mental Life, both of its phenomena and its conditions. The phenomena are such things as we call feelings, desires, cognitions, reasonings, decisions, and the life; and, superficially considered, their variety and complexity is such as to leave a chaotic impression on the observer.” (James, 1890/1950, p. 1).

Herein lies a cornerstone of what we call situated psychology, namely the articulation of a relationship between ‘mental life’ and its conditions. It is, in our view, the relationship between ‘mental life’ and its conditions that situated psychology should revolve around. Let us proceed by considering how James understood so-called ‘mental life’ and its ‘conditions’.

James wrote in *Principles* that it should be understood vaguely; on the one hand, it included both ‘mental states,’ and on the other hand, he explicitly considered whether our automatic and instinct-like habits should also be subject to analysis (James, 1890/1950, pp. 5–6). In classic functionalist terms, he argued that the primary criterion for the mental must lie in the pursuit of goals by means of ends: “The pursuance of future ends and the choice of means for their attainment are thus the mark and criterion of the presence of mentality in a phenomenon.” (James, 1890/1950, p. 8, italics in the original).

Thus, mental life itself, according to James, can be understood broadly and concretely, but still with a kind of goal-directedness as its central criterion. It should be briefly mentioned here that James in *Principles* was explicitly dualist, although he later wrote that this was mostly a pragmatic attempt to keep metaphysics and psychology separate, and that such a separation was probably impossible (see Heft, 2001, p. 19; Leary, 2018, pp. 53–55). James’ career after the publication of *Principles* can be seen as an attempt to explicitly formulate a philosophical and metaphysical basis for psychology that is fundamentally anti-dualistic and could theorize experience. This philosophical direction, which he later ended up calling radical empiricism, will not be introduced here, but we will highlight some of James’ later thoughts because they further elaborate on the understanding of mental life.

In the article ‘Does Consciousness Exist’ (1904), James explicitly revised his earlier dualism and proclaimed that consciousness does not exist; that his dualistic approach in *Principles* was a pragmatic necessity rather than an expression of his good will; and that what we call ‘mind’ as well as what we call ‘body’ must be made out of one substance—‘pure experience.’ This was James’ attempt to transcend Cartesian dualism (wherein ‘the mental’ is seen as consisting of one substance, while ‘the material’ is seen as consisting of another). The relationship between thinking and thing, between the knower and what is known, is thus characterized by a relational connectedness rather than a separation (see Heft, 2001, pp. 27–28; James, 1904). “That entity [consciousness] is fictitious, while thoughts in the concrete are fully real. But thoughts in the concrete are made of the same stuff as things are.” (James, 1904, p. 491). Consciousness—or the mind—is not an entity but rather a function, a process. As he wrote in another article, “The fundamental fact about experience […] is that it is a process of change.” (James, 1909/2000, p. 162).

In other words, this loose idea of ‘mental life’ as fully real in its processual concreteness allows for a pluralistic and ideographic investigation of all kinds of experiences. Feelings, thoughts, hopes, but also hallucinations, imaginations, dreams—all must be considered real, but not necessarily true. Mental life, according to James, should thus not be understood as static entities located inside people’s heads, but rather as a kind of processual capacity.

If mental life, in our reading of James, should thus be understood broadly and as a process how should its conditions be understood? One of his early examples in *Principles* was memory. Why do we, he asked, remember things from our childhood better than what happened an hour ago? And, he added, why does illness affect our ability to remember? Memory, he wrote, “does not exist absolutely, but works under conditions; and the quest of the conditions becomes the psychologist’s most interesting task.” (James, 1890/1950, p. 3). In other words, if we understand mental life as a processual capacity, then this capacity has several *physiological* conditions that set (some of) its boundaries and possibilities. James himself remarks that the hands’ abilities to feel, and the eyes’ ability to see, are clear conditions for the specifics of mental life (James, 1890/1950, p. 4). But these conditions should, in our view, not be limited to the physiological. For as we saw above, James was precisely concerned with the directedness of the psychological, and he described this directedness as shaped by our surroundings: “minds inhabit environments which act on them and on which they in turn react” (James, 1890/1950, p. 6). The study of “mind in the midst of all its concrete relations” (James, 1890/1950, p. 6) was, according to Heft (2001, pp. 17–18), a central idea for James.

This early formulation of psychology as the science of mental life and its conditions should, in our view, be understood in light of James’ later philosophy, where he emphasized the relational and the active (and, arguably, some of James’ later ideas were already subtly present in *Principles (*see Leary (2018, p.54).

The conditions of mental life must therefore be understood as its concrete relations—including the body—as here in Heft’s presentation of James: “Fundamentally and concretely, we are active beings. We move around and act on environmental features. We experience the environment through our bodies, and reciprocally, we experience our bodies through engaging the environment.” (Heft, 2001, p. 55). This fits, of course, with James’ Darwinian inspiration where, as Leary (2018, p.18) has usefully put it, “mental life serves the purpose of the organism”.

We can thus understand the conditions of mental life as the conditions of the environment (where can we go?), as well as the physiological and neurological conditions of the body.

As should hopefully be clear by now, James’ understanding of mental life differs quite significantly from mainstream psychological research, both the research at his time and much of the research done through the 20th and early 21st centuries. James laid the groundwork for an understanding of mental life as being bodily anchored, processual, and characterized by activity. We will thus argue that James’ ideas fundamentally express a situated understanding of psychology because he emphasizes mental life as being precisely an active and processual part of the world. Mental life, in a Jamesian view, is not stale ‘processing’, but rather a series of diverse, pluralistic and *concrete* phenomena that arise *in the world* and which are not locked away in a cartesian mind-vault. Mental life, in this understanding, is characterized by a fundamental dependency—both on the body and its organs, but also on the social, the material, and the relational.

As we will see in the following sections, the question of the conditions under which people live is a central question for several psychological traditions. In the following, we will move from James’ ideas to a more contemporary—and somewhat different—understanding of the question of the situated, namely from situated learning theory.

## Situated Learning and the Understanding of the Situated

In his dissertation *Musical Apprenticeship: Learning at the Academy of Music as Socially Situated*, the Danish professor of psychology, Klaus Nielsen, argues that the Russian (cultural) psychologist and activity theorist, Alexander R. Luria, was one of the first within psychology to theorize the concept of situatedness (Nielsen, 1999). Based on Luria’s empirical studies of cognitive and categorization processes among peasants from both traditional and modern agriculture in Uzbekistan in the 1930s (published under the title *Cognitive Development: Its Cultural and Social Foundations* in 1976), Luria formulates a distinction between “situational” thinking and “abstract” thinking (Luria, 1976, pp. 54–55).

In Luria’s (1976) experimental study, the participants are shown four pictures of tools, three of which are clearly placed within a well-defined category and the fourth is not (e.g., hammer, saw, hatchet and log, (Luria, 1976, p. 55). The task for the participants is to select the three tools that belong together. The peasants from the modern agricultural practices choose the three tools that belong to the same category (hammer, saw and hatchet). This pattern of selection Luria calls ‘abstract category.’ But the peasants from the traditional agricultural practices choose first all pictures, afterwards three other pictures as belonging to the same category (hammer, saw, and log). The logic in their understanding of the connection is tied to experienced, concrete, practical situations where the three objects can be used together: “They’re all alike. The saw will saw the log and the hatchet will chop it into small pieces. If one of these things has to go, I’d throw out the hatchet. It doesn’t do as good a job as a saw” (Luria, 1976, p 60).

Luria theorizes that the groups’ different ways of categorizing are due to the fact that they are participants in different types of activities with different ways of understanding the world. One group has a concrete and practical understanding of reality, i.e. they operate “on the bases of a “practical utility”” (Luria, 1976, p.59). The other group has an abstract and linguistically logical understanding of reality. Regarding the concrete, practical way of experiencing reality, Luria uses the term ‘being situated’ (Luria, 1976; Nielsen, 1999).

In Luria’s presentation of knowledge as situated in the example above, a distinction is made between practical thinking (as related to a practical and everyday relationship to tools) and philosophical thinking (as related to a (linguistically) abstract relationship to tools); and it is only the one type of knowledge—the practical one —that is understood as situated. It is precisely this distinction between situated/concrete knowledge and abstract/general knowledge that the anthropologist Jean Lave transcends through her theory of Situated Learning.

The theory of Situated Learning originates from Lave’s studies of the Vai and Gola tailors’ learning processes in Liberia in the 1970s. Through these analyses and later empirical studies, for instance of arithmetic skills associated with calculating discounts in a supermarket, Lave develops an understanding of knowledge (and thus of learning) as ‘situated’: “Learning, knowledgeability, skillfulness, whatever else might be, are always only part of ongoing social arrangements and relations” (Lave, 2011, p. 3). For Lave the central point is to transcend the fundamental distinction and dualism between the situated and the abstract found in Western culture (Lave & Wenger, 1991). The point is that learning and knowledge are always “embedded in social conditions (family size, habits, hierarchy, organization, etc.) rather than solely being embedded in cognitive structures” (Nielsen, 1999, p. 15). This means that learning is intertwined with a wide range of social relationships in everyday life; learning is thus a situated phenomenon, understood as a dialectic between participating people and the contexts (practices) in which people’s activities are shaped (Lave, 1988).

To transcend the dualism between the situated and the abstract/general, Lave develops a relational theory of practice, which has roots in Marx’s understanding of praxis, where the concepts of ‘everyday life’ and ‘participation’ are central (Lave & Packer, 2008; Packer & Goicoechea, 2000). We will therefore turn to a description of the concepts of practice, everyday life, and participation.

## Practice

Marx’s concept of praxis simultaneously draws from and breaks with Hegel’s dialectic. Based on the American philosopher Richard J. Bernstein’s (1971) reading of Marx, our relationship to the world must first and foremost be understood as a practice relationship, as opposed to a knowledge relationship (Bernstein, 1971, p. 35). This understanding of dialectic means that for Marx, it is the human-produced changes in the world that shape and change our thinking, and not the other way around. The value of philosophy is thus deeply connected to the production of change (cf. Marx’s concept of revolution) according to Marx (Avineri, 1968).

Lave (2009) develops her understanding of the situated nature of cognition and learning based on Marx’s philosophy of praxis, which Lave elsewhere calls a social ontology (Lave & Packer, 2008). She emphasizes that it is not “knowledge that develops social life or is social life, but rather praxis, the production and action of social life, that produces knowledge in change as part of ongoing practice” (Lave, 2009, p. 16). This form of philosophy “sees practice as the dialectical relationship we have in the human world,” as the Danish philosopher Thisted (2015, p. 112) describes it. Practice is precisely the concept for the dialectical relationship between philosophy (ideology) and material, historical reality (practical activity) and thus a break with philosophy’s separation of theory and reality.

Using the American philosopher Andrew Feenberg’s (1981) concept, we can understand Marx’s philosophy as a ‘philosophy of practice’. With this basis, we must therefore not misunderstand practice as an opposition to theory. Practice is the union. In practice—the actual life that people live—there is an inherent understanding, interpretation, or ‘theory’; i.e. a *situated* understanding.

The Norwegian professor of psychology, Steinar Kvale, wrote in 1964 very accurately about this understanding of human practice, which places the “concrete, real human being at the center. It lives in a specific historical situation and in a specific social relationship to its fellow human beings. Human activity is primarily practical activity” (Kvale, 1964, p. 195). Thus, we cannot separate the reality that we recognize from the practical process or activity of recognition as Thisted (2015,) claims. He adds that Hegel’s concept of spirit is replaced by Marx’s concept of praxis, which precisely denotes this dialectical relationship between human and world (Thisted, 2015).

The concept and emphasis on the situatedness of human being in Jean Lave’s work can further be said to highlight this movement’s attempt to transcend the human-world dualism; spirit-praxis-situatedness.

## Everyday Life and Participation

The concept of everyday life has a long history and is often described as a particular temporal, material/situational, and social aspect of people’s lived lives. The temporal aspect concerns the daily, repetitive, and cyclical nature of everyday life (Felski, 1999). For instance, Lefebvre (1987) writes that understanding people’s everyday practices involves gaining knowledge of its cyclical structure. Winther (2017) adds a central dimension to the way everyday life is often theorized—that everyday life *takes place*—and thus points to a particular situational and place-specific aspect of the concept of everyday life.

In the chapter *Towards A Social Ontology of Learning*, Lave and Packer (2008) point out that the concept of everyday life is often linked to a question of where everyday life is located (location). But such a conceptualization risks maintaining the dualism between everyday life (practice) and philosophy (ideology) that the philosophy of praxis precisely seeks to transcend. Lave and Packer’s (2008) understanding of the concept of everyday life is therefore more closely tied to the philosophy of praxis: “For the Western Existential Marxists philosophers, knowing consists of raising everyday conditions to consciousness through reflection, against the present historical horizon” (Lave & Packer, 2008, p. 41).

This understanding is based on a social and historical ontology where everyday life (practice) does not stand in a dualistic relationship to the special, abstract, reflective (philosophy, ideology), but is understood as a dialectical relationship between being and knowledge (Lave & Packer, 2008, p. 42). Lave and Packer (2008, p. 42) write, based on the philosophy of praxis, that “the Marxist project (…) grasps the everyday as a region of poesis—a site of the practical reproduction of persons and society.”

The discussion around the concept of everyday life further emphasizes the understanding of situatedness within Situated Learning theory because it helps to nuance the understanding that ‘situated’ refers to more than just being in particular places, with particular others, at particular times. It is also a certain way of understanding our being in the world. That is why Lave and Packer (2008) use the concept of everyday existence. And in the same way, the concept of participation becomes central in Situated Learning. Participation is used to emphasize the person as an active agent in practice: Bernstein (1971, p. 44) summarizes the significance of human participation: “Consequently the very nature or character of a man is determined by what he does or his praxis.” The participating actor is dialectically constituted by and constitutive of practice and its conditions. People participate in everyday life with their bodies, in interaction with other people, and use material artifacts in relation to what is meaningful in the specific context and in relation to the individual’s desires and interests (Holland & Lave, 2019). The concept of participation thus points to a situated understanding of persons-in-practice, where the person and practice are dialectically connected through participation.

In summary, a situated understanding based on Situated Learning is a (theoretical) insistence that human activity can only be understood with insight into the practices in which people’s lives are embedded and unfold.

## Situated Psychology as Psychological Externalism

The idea of the situated, as we have laid it out in the preceding sections, emphasizes that human activity takes place in concrete situations and with concrete others. These activities, crucially, helps constitute the very situation they are placed in. Human activity is thus both framed by specific circumstances that precede it and help to create (and potentially transform) those same circumstances. But if this leaves us with an understanding of the situated, what about the psychological? How should we approach the psychological—which we here understand broadly and pluralistically as thoughts, feelings, dreams, expectations, worries, reason, and so on—without reintroducing the dualism that Lave seeks to escape?

To do this, we will introduce and discuss the idea of psychological externalism, originating in philosophy, in contrast to Lave’s anthropological perspective and James’ psychological perspective. Psychological externalism starts with the question of how we should understand the psyche. Is the psyche merely what the brain does? Or is the psyche perhaps extended, as philosophers Andy Clark and David Chalmers have become famous—and infamous—for claiming (Clark & Chalmers, 1998)? Psychological externalism thus offers yet another way to understand situated psychology, which is elaborated below. We start with a general understanding of externalism and then work our way towards an understanding of psychological externalism’s contribution to situated psychology.

## Externalism Generally Understood

Externalism asserts that a large part of our psychological processes depends on our relationship with the environment (Roberts et al., 2019). That is, in an externalist position, psychological processes can only be meaningful insofar as we have relationships with the environment. This is in contrast to an internalist position, which would claim that psychological processes are primarily to be understood as based on conditions that belong to the structure of our consciousness, such as the neurological structure of consciousness.

Different positions within externalism exist. These positions generally depend on questions about what kind of relationship we have with the environment and what kind of environment, or content, our psychological processes depend on. The question of what kind of relationship we have with our environment can be understood on a spectrum. Some argue that these relationships should be understood as pure causal relationships with the environment, such as a stimulus-response relationship, while other philosophers also understand it as norm-based behavior (Burge, 2007; Davidson, 2001).

We can distinguish here between explicit naturalistic and normative understandings of the relationship, and thus also what in the environment is emphasized. Is it relationships with the natural environment, or are social ontological issues involved, with norms, rules, and institutions playing a crucial role? Many externalist positions attempt to combine these understandings, so the environment is seen as both natural and social, thus avoiding introducing a new dualism between the natural and the social (Costall, 1995). As Arnau et al. (2013, p. 4) claim externalism can be seen as providing a significant part of the theoretical background of many recent developments within the understanding of cognition, like distributed cognition (see for example Hutchins, 1995), enactive cognition (see for example Thompson, 2007) and extended cognition, which we will address below.

Externalist positions, despite their internal differences, can all be said to be anti-dualistic. They oppose both explicit and implicit understandings that involve seeing ‘the mental’ and ‘the material’ as two separate substances. As we will see in the next section, a particular variant of externalism has gained attention in 4E cognition, namely the thesis of the extended mind.

## The Thesis of the Extended Mind

The thesis of the extended mind (see Menary 2010) is the idea that psychological processes are not only internal to each organism but can be said to exist in the use of artifacts, tools, and so on. In this perspective, psychological processes are not just ‘in the head’ but also outside of it. Let us provide a brief overview following Cash’s (2013) description of the three phases in the development of the thesis so far.

The first phase consists, as mentioned, of Clark and Chalmers’ (1998) original question and answer: “Where does the mind stop and the rest of the world begin?” (Clark & Chalmers, 1998, p. 7). For Clark and Chalmers, three possible answers exist: (1) A standard assumption that what is outside the body is outside the mind, (2) an externalism focused on meaning, and (3) active externalism, which is Clark and Chalmers’ position. Their original formulation of the thesis is that certain objects and things while being separate from us can still function as part of our cognitive processes. On that basis, Clark and Chalmers propose what they call a parity principle: “If, as we confront some task, a part of the world functions as a process which, were it done in the head, we would have no hesitation in recognizing as part of the cognitive process, then that part of the world is (so we claim) part of the cognitive processes. Cognitive processes ain’t (all) in the head” (Clark & Chalmers, 1998, p. 8).

For Clark and Chalmers, there is a form of functional parity between the cognitive processes and the external things that surround us and can help us in those processes. Their famous example of this asks the reader to imagine two people, both of whom want to visit the Museum of Modern Art in New York. One person, whom they call Inga, has a well-functioning memory, and she can remember the address of the museum, she knows where it is, and it is easy for her to get there. Otto, on the other hand, has Alzheimer’s, and has a detailed notebook that he often consults in his daily activities in order to help himself. When he decides to visit the museum, he looks up the address and route in his notebook, where it is neatly written down. He thus cannot remember the address without help from his notebook. Clark and Chalmers’ point is that there is practically no difference between Otto and Inga: “…the notebook plays for Otto the same role that memory plays for Inga. The information in the notebook functions just like the information constituting an ordinary non-occurrent belief; it just happens that this information lies beyond the skin.” (1998, p. 13).

Both the notebook and the memory play a causal role in both Otto and Inga’s behavior. Clark and Chalmers’ idea has only become more relevant and perhaps convincing with the advent of smartphones, giving us access to all kinds of information instantly. Thus, a contemporary example is that few people worry about remembering phone numbers anymore, with only very few numbers being literally kept in mind. The rest we leave to our mobile phones to remember for us, and they thus become part of our cognitive processes involving memory.

Despite Clark and Chalmers’ attempt to extend psychological processes to include the environment, these processes are still modeled on processes that happen “inside the head.”. That is to say, the processes which Clark and Chalmers imagine become *extended* are the processes that were internal before (e.g. memory).

As Cash (2013, p. 62) points out, this creates a very individualistic starting point for Clark and Chalmers, which later phases attempt to overcome. Furthermore, it also seems that a consequence of the parity principle is that cognitive processes can be extended in a limitless manner without regard to what they are extended with (Clark & Chalmers, 1998, pp. 16–18). In principle, any person one talks to, any book one reads, and any websites one visits could all be said to be part of the cognitive processes. And this is where the second phase comes in. Clark and Chalmers’ paper has been both provocative and influentialand has spurred a significant series of discussions and developments which we cannot delve into here (but see, for example, Cash, 2013; Chemero, 2009; Gallagher, 2013). One critique (Cash, 2013, p.63) has been that the idea of the extended mind is still rather *individualistic* – it decouples psychological processes (such as remembering) from their socio-cultural context. Thus, more recent developments, often referred to as 4E cognition, include a greater emphasis on how cognition is “socially and culturally distributed” (Cash, 2013, p.63).

The idea here is that a socio-cultural practice and institution like the legal system enables specific cognitive processes. An example from Gallagher concerns making legal judgments. It requires both an active relationship between individuals, the use of language as a tool, and the legal system as an institution (Gallagher, 2013) sanctioning whatever judgments are made. The legal system is, according to Gallagher (2013), both a product of the interaction between individuals, tools (e.g. language, IT-systems), and specific socio-cultural practices (handling of juries, questioning of witnesses etc.). However, the legal system also functions as a resource that extends and transforms the interacting individuals’ cognitive processes, even though it itself is a product of, among other things, these individual’s own cognitive processes. The cognitive processes taking place in the legal system should therefore be understood as distributed—they do not belong to the individual alone, at least not exclusively, but are rather distributed over many different people, legal practices, artifacts such as legal texts, etc. Unlike the image of cognition in the first phase, the cognitive processes here occur just as much from the outside in as from the inside out.

We can now describe the concept of internalism that is sought rejected more closely. Most proponents of 4E see the ‘classical’ model of cognition as one wherein cognition is a syntactically based manipulation of representational mental structures which happens ‘internally’ to the organism (Newen et al., 2018, p. 5). In the internalist perspective, in other words, cognition is a process which mediates between the sensory input from our surroundings and the motor outputs that result in various actions. A classic example of the internalist understanding is the work by the psychologist Kosslyn (1981). His research in the 1980s focused on imagination. How is it that so many people have an experience of being able to ‘see’ images with their ‘inner eye’? How can that ability be explained? Kosslyn’s explanation for this was that our ‘input’ (what we see, for example, a car) is mentally represented in a kind of ‘quasi-image’ (Kosslyn, 1981; Kosslyn et al., 1978), containing a number of the same qualities as the object itself (the ‘quasi-image’ of your car would thus contain the same color, number of wheels, leather upholstery, etc. as your real car). It is these mental representations and their processing that can then lead to motor action, for example, when you change gears while driving without looking at the gear stick. Cognition here is an isolated and relatively abstract process that primarily occurs in the brain. But it is precisely the above ‘internalist,’ isolated, and abstract understanding of cognition that is too narrow according to proponents of 4E cognition. They claim instead:“…the cognitive phenomena that are studied by modern cognitive science, such as spatial navigation, action, perception, and understanding others’ emotions, are in some sense all dependent on the morphological, biological, and physiological details of an agent’s body, an appropriately structured natural, technological, or social environment, and the agent’s active and embodied interaction with this environment.” (Newen et al., 2018, p. 5).

As the attentive reader will notice, this modern definition is surprisingly close to the thoughts from James, which we previously described, as well as to the perspectives from situated learning. We have already emphasized cognition as embedded above—that is, the whole idea of situatedness as implying psychological processes as related to and conditioned by the temporal, spatial, and socio-cultural frameworks in which they occur. We will briefly describe the remaining two E’s: Enactive (loosely understood as active) and embodied (loosely understood as corporeality).

With a focus on corporeality, 4E cognition emphasizes that the brain is not the only basis for cognitive processes; rather, cognitive processes should be understood as stretched between brain-body-environment. As Crippen and Schulkin (2020) describe it, we should understand this ecologically since it is the organism’s bodily interaction with the environment that helps structure and constitute the organism’s perception and cognition. This is (again) similar to James’ claim that the mental life is, of course, to some extent dependent on a number of physiological conditions—cognition and perception are, for example, dependent on the body’s ability to orient itself, the eyes’ ability to see, and so on. The overall question the turn to embodiment revolvers around is, somewhat simplified, a question of to what extent—and how—cognition is dependent on the manifold bodily processes (Newen et al., 2018).

With a focus on the active (enactive), we move into another aspect, namely that cognition should be seen as the process of actively being involved in and with the different kinds of environments in which the cognitive process in question is embedded, rather than being passively receptive to various ‘inputs’.

The active aspect thus emphasizes the cognitive process as connected to abilities or dispositions to act. This can be compared with ecological psychology, where, for example, Gibson (1979/2015) emphasized perceptual processes not as something passive that happens to us but is active and something done by being related to the actions and circumstances where the perception takes place.

Overall, 4E cognition is thus characterized by a relational understanding of cognitive processes: cognition does not only occur within the confines of the skull but through the whole body and its dispositions to interact with environments consisting of material and social elements, and through the different situations where these interactions are embedded. Cognition is here, in our understanding, a dynamic relational process and a process that is experience-based, i.e., 4E cognition is characterized by processes that can be both conflictual (Aagaard, 2021) as well as harmonious. For example, Aagaard (2021) has argued that technology can indeed extend our cognitive abilities, but it is not necessarily positive: a smartphone can enable us to remember and find our way, but also to become distracted! To summarize and conclude, 4E cognition should be understood as a broad field (with multiple discussions and disagreements) which provides a broadly situated approach to the psychological.

## Situated Psychology as Historically Situated

The last point we will argue is that these situated psychological processes must be understood as historically situated. The question of psychological processes and history is, of course, classic—and should here be understood as separate from the question of the history of psychology as a discipline. Kenneth Gergen’s famous article from 1973, “Social Psychology as History,” argued, for example, for the (then) radical claim that social psychology was not a natural science and could not demonstrate universal laws of behavior. For Gergen, social psychology was instead an investigation of historical conditions (Gergen, 1973, p. 310). For example, he argued that Milgram’s famous obedience experiments were a result of the contemporary relationship to authority (Gergen, 1973, p. 315). In other words, a number of classic psychological processes—such as obedience—must to some extent, according to Gergen, be dependent on specific societal (and thus historical) processes. As he described it:

“Social phenomena may vary considerably in the extent to which they are subject to historical change. Certain phenomena may be closely tied to physiological givens […] We must think, then, in terms of a continuum of historical durability, with phenomena highly susceptible to historical influence at one extreme and the more stable processes on the other.” (Gergen, 1973, p. 318).

A few years later, the historian of psychology Kurt Danziger argued for something even more radical, namely that the essence of psychological phenomena is that they have a historical variation: “…the essence of psychological categories (insofar as they have one) lies in their status as historically constructed objects.” (Danziger, 1997, p. 12). For Danziger, this means that it is not only the methods, ideas, theories, and concepts of psychology that change over time, but that the psychological itself is historically variable: “Human subjectivity, the reality behind the objects of psychological investigation, is itself strongly implicated in the historical process, both as an agent and as a product.” (Danziger, 1994, p. 475).

A similar idea is found in the psychologist Philip Cushman, who has argued that psychology is historically situated (Cushman, 1990). For Cushman, this specifically means that the self is dependent on specific historical developments. His examples are the Victorian and sexually repressed self, contrasted with the post-war so-called “empty self,” which according to Cushman arises in the wake of the consumer society, its lack of community, and its focus on “self-improvement” (Cushman, 1990, p. 604). The details of Cushman’s analysis, which primarily focuses on the USA, are less relevant for this article. More relevant is that Cushman here inscribes himself in a series of thinkers who, in one way or another, articulate a series of ideas about how the psychological is constituted by historical processes.Similar arguments can also be found in ecological psychology, where, for example, Edward Reed many years ago argued that perception was a historical process (Reed, 1986), Birk & Manning, (2023) have argued that this must be understood in two ways:

1. First, what can be perceived is historically constituted through material, social, economic, political, and interactional processes. It is a series of complex processes that have determined what we see—a housing complex or a forest, for example.
2. Second, perceptual (and cognitive) processes are historical in an ontogenetic sense: our perceptual abilities develop from infancy to adulthood, both biologically (newborns, for example, have not yet developed the ability to see significantly) but also normatively—we learn to perceive the meaning in what we see (Birk & Manning, 2023; Heft, 2017a).

The American historian of biology Landecker (2016) has pointed out that history has a biology, in the sense that historical, industrial, and capitalist processes have transformed both humans and other organisms, for example, in the development of antibiotics, which changed both humans and microbes (e.g., the development of antibiotic-resistant bacteria, etc.). Analogously, one might say that if history has a biology, then history also has a psychology, in the sense that specific psychologies—specific ways of thinking, feeling, understanding, etc.—have arisen through historical developments (Brinkmann, 2008). These historical developments also include the emergence of psychological science, which has greatly influenced human psychology. The same idea is put forward by the historian of psychology Graham Richards:

“The historian of Psychology is not only looking at the history of a particular discipline, but also at the history of what that discipline purports to be studying […] ‘Doing Psychology’ is the human activity of studying human activity; it is human psychology examining itself—and what it produces by way of new theories, ideas and beliefs about itself is also part of our psychology!” (Richards, 2009, p. 7).

In a slightly different context, the philosopher Kim Sterelny has argued that cognitive processes are scaffolded by technologies and institutional contexts that support and shape these processes. As an example of his point, he uses eating and digestion. The ways humans eat are obviously supported by a wide range of technological processes that process food in all sorts of ways, making it easy to find (e.g., in the supermarket) and easy to digest. Where other animals, such as cattle, have to spend hours digesting what they eat, human ability to process food means we do not need this—we can cook the meat, grind the grain and bake bread, cook rice, and so on. As Sterelny writes:

“We have engineered our gustatory niche; we have transformed both our food sources and the process of eating itself. Our under-powered jaws, short gut, small teeth and mouth fit our niche because we eat soft, rich and easily digested food. Our digestive system is environmentally scaffolded.” (Sterelny, 2010, p. 468).

Sterelny, Richards, Danziger, Landecker, and Gergen do not have much in common. But across their works, two overarching arguments emerge:

1. First, a question about the nature of psychological processes. These thinkers all emphasize that psychological processes have a nature that might best be described as “interactive” (Hacking, 1999) and recursive. Here, different psychological processes are transformed through the historical and situated, through the relationship to materiality, technology, tools, and institutions, including through the encounter with psychological ideas.
2. Second, it is a recurring argument that the historical is precisely these material, technological, institutional processes. In other words, when we have written about the situated in this article, it is important to remember that this situatedness also has a historical character. Historical processes are precisely the use of tools, interactions, practices, and institutional contexts (among others). Historical processes do not run ‘alongside’ the psychological as a kind of separate area. ‘History’ is rather immanent in both the use and development of tools and institutions, and any focus on the situated should therefore also have an eye for these contexts.

## Conclusion

The idea of situated psychology contains two elements: the question of psychology and the question of the situated. The approaches we have reviewed understand both the situated and the psychological somewhat differently and with different emphases. The differences from James to Lave to Clark to Danziger, etc., are many. These are different ideas, developed at different times (and for different purposes) and with different focuses, but these ideas have some common features that we see as characteristic of situated psychology, and it has been the aim of the article to focus on these common features.

If James’ radical empiricism is an early suggestion of how psychology can develop in a **non-dualistic** direction, but with a primary focus on the individual, then Lave’s practice-theoretical understanding gives us a suggestion of how psychological phenomena can be understood as situated in people’s concrete and **practical activities**. The externalist view, represented by scholars such as Clark, Chalmers, Cash and Gallagher gives us a suggestion for how the psychological dimension of the situated can be understood as something that in a radical sense exists **in the world.** Lastly, this sense of the psychological existing concretely in the world is also **historical**, insofar as the past “lives on” via artefacts, materials, buildings, rituals, habits, and so forth. What we have pointed out above through discussions of James, Lave, and the externalist position is that they all point to psychological phenomena as situated phenomena, but also in a more radical sense psychological phenomena are not ‘in the head’. As the psychologist Harry Heft has written, this is an idea that:

“…psychological processes cannot be justifiably seen as events occurring within the boundaries of the body in any simple sense. Instead, the realm of the psychological encompasses the environment (defined relationally) to include, among other things, tools, artifacts, and representations” (Heft, 2001, p. xxxiv).

The psychological is understood here as something that, in one way or another, exists in the world, rather than exclusively inside the body or head. This is, broadly speaking, what we have tried to argue throughout the article: that the situated in situated psychology should be understood as a focus on the concrete and material processes through which people’s daily lives unfold, and in that practice, simultaneously create and co-create the frameworks for their own unfolding. Therefore, the psychological does not only concern individuals with various thought processes ‘inside the head,’ who co-create the circumstances they live under. It is rather the situated practice itself—including, for example, the understanding of persons and the use of artifacts such as notebooks, computers, phones, etc.—that is psychological. Understood in this way, the psychological also includes the tools and instruments that we use in all sorts of ways in our daily lives, and the environments we are part of.

And precisely for this reason, a central premise for situated psychology is that ‘the psychological’ cannot be understood in isolation from the **historical and social circumstances** in which people’s lives are embedded. All the authors of this article may think in Danish, but that psychological fact is obviously given to us from our social and historical embeddedness in Danish society, Danish relationships, Danish language, and so on. That part of our psychological make-up, so to speak, is thus situated in a series of local circumstances.

The psychological may be understood/experienced in many cases as ‘private’ or ‘personal’—for example, our experience of inner life or thoughts—but it is simultaneously a phenomenon that is situated in the world in a relational, cultural, material, and historical sense.

Our point with the article has not been to sketch a stringent theoretical model, but rather to sketch an overarching theoretical and intellectual framework for situated psychology, and to underline that there is a significant intellectual history that broadly supports these ideas. However, despite the many similarities in the four different perspectives we have outlined (e.g., the ambition to be anti-dualistic), there are also significant differences in how the material, social, relational, etc., should be understood, including methodological differences.

For example, one can discuss to what extent the situatedness of the psychological is actually situated. Should we, as Bruno Latour once argued (Latour, 1996) when he read Hutchins’ ‘Cognition in the Wild’ (Hutchins, 1995), move all the psychological out of the head? Does one believe, like Clark and Chalmers, that psychological processes actually take place in the notebook or computer? Or does one rather believe that these processes are scaffolded (Sterelny, 2010) by the world in all its concrete diversity?

We will conclude our article with these open questions, which call for further theoretical, methodological, and empirical development.

## Data Availability

No datasets were generated or analysed during the current study.

## Footnotes

1 We would like to thank the reviewers for making this astute connection.

## References

[CR1] Aagaard, J. (2021). 4E cognition and the dogma of harmony. *Philosophical Psychology*, *34*(2), 165–181. 10.1080/09515089.2020.1845640

[CR2] Angell, J. (1907). The Province of functional psychology. *Psychological Review*, *14*(2), 61–91. 10.1037/h0070817

[CR3] Arnau, E., Estany, A., del González, R., & Strurm, T. (2013). The extended cognition thesis: Its significance for the philosophy of (cognitive) science. Philosophical Psychology, 27(1), 1–18.

[CR4] Avineri, S. (1968). *The social and political thought of Karl Marx*. Cambridge University Press. 10.1017/CBO9781139171410

[CR5] Bernstein, R. J. (1971). *Praxis and action*. University of Pennsylvania.

[CR6] Birk, R., & Manning, N. (2023). Towards an ecological social science? On introducing ‘social affordances’ to (some) social theory. *Philosophical Psychology*. 10.1080/09515089.2023.2277346

[CR7] Blumer, H. (1954). What is wrong with social theory? *American Sociological Review*, *18*, 3–10.

[CR8] Brinkmann, S. (2008). Changing psychologies in the transition from industrial society to consumer society. *History of the Human Sciences*, *21*(2), 85–110. 10.1177/0952695108091412

[CR9] Brinkmann, S. (2009). Hvad er psyken? Almenpsykologiske og forskningsmetodologiske perspektiver. Professortiltrædelsesforelæsning ved Institut for Kommunikation, AAU. https://vbn.aau.dk/da/publications/hvad-er-psyken-almenpsykologiske-og-forskningsmetodologiske-persp

[CR10] Brinkmann, S. (2011). Dewey’s neglected psychology: Rediscovering his transactional approach. *Theory & Psychology*, *21*(3), 298–317. 10.1177/0959354310376123

[CR11] Burge, T. (2007). *Foundations of mind: Philosophical essays* (Vol. 2). Oxford University Press.

[CR12] Cash, M. (2013). Cognition without borders: Third wave socially distributed cognition and relational autonomy. *Cognitive Systems Research*, *25–26*, 61–71. 10.1016/j.cogsys.2013.03.007

[CR13] Chemero, A. (2009). Radical Embodied Cognitive Science. Bradford.

[CR14] Clark, A. (1999). An embodied cognitive science? *Trends in Cognitive Sciences*, *3*(9), 345–351. 10.1016/S1364-6613(99)01361-3 10461197 10.1016/s1364-6613(99)01361-3

[CR15] Clark, A., & Chalmers, D. (1998). The extended mind. *Analysis*, *58*(1), 7–19.

[CR16] Costall, A. (1995). Socializing affordances. *Theory & Psychology*, *5*(4), 467–481. 10.1177/0959354395054001

[CR17] Costall, A. (2004). From Darwin to Watson (and Cognitivism) and back again: The principle of Animal-Environment mutuality. *Behavior and Philosophy*, *32*(1), 179–195. JSTOR.

[CR18] Crippen, M., & Schulkin, J. (2020). Mind ecologies: Body, brain, and world (pp. ix, 321). Columbia University Press. 10.7312/crip19024

[CR19] Cushman, P. (1990). Why the self is empty. Toward a historically situated psychology. *The American Psychologist*, *45*(5), 599–611. 10.1037/0003-066X.45.5.599 2190505 10.1037//0003-066x.45.5.599

[CR20] Danziger, K. (1994). Does the history of psychology have a future?? *Theory & Psychology*, *4*(4), 467–484. 10.1177/0959354394044001

[CR21] Danziger, K. (1997). *Naming the mind: How psychology found its Language*. SAGE.

[CR22] Davidson, D. (2001). *Subjective, intersubjective, objective: Philosophical essays volume 3*. Oxford University Press. 10.1093/0198237537.001.0001

[CR23] Dewey, J. (1896). The reflex Arc concept in psychology. *Psychological Review*, *3*(4), 357–370. 10.1037/h0070405

[CR24] Feenberg, A. (1981). *Lukács, marx, and the sources of critical theory*. Oxford University Press.

[CR25] Felski, R. (1999). The invention of everyday life. New Formations, 39.

[CR26] Gallagher, S. (2013). The socially extended mind. *Cognitive Systems Research*, 25–26. 10.1016/j.cogsys.2013.03.008

[CR27] Gergen, K. J. (1973). Social psychology as history. *Journal of Personality and Social Psychology*, *26*(2), 309–320. 10.1037/h0034436

[CR28] Gibson, J. J. (2015). *The ecological approach to visual perception*. Psychology. (Original work published 1979).

[CR29] Hacking, I. (1999). *The social construction of what??* Harvard University Press.

[CR30] Heft, H. (2001). *Ecological psychology in context: James gibson, Roger barker, and the legacy of William james’s radical empiricism*. Psychology. 10.4324/9781410600479

[CR31] Heft, H. (2017a). Perceptual information of an entirely different order: the cultural environment in the senses considered as perceptual systems. *Ecological Psychology*, *29*(2), 122–145. 10.1080/10407413.2017.1297187

[CR33] Holland, D., & Lave, J. (2019). Social practice theory and the historical production of persons. In A. Edwards, M. Fleer, & L. Bøttcher (Eds.), Cultural-historical approaches to studying learning and development: Societal, institutional and personal perspectives (pp. 235–248). Springer. 10.1007/978-981-13-6826-4_15

[CR34] Hutchins, E. (1995). *Cognition in the wild*. MIT Press.

[CR35] James, W. (1904). Does ‘consciousness’ exist?? *Journal of Philosophy Psychology and Scientific Methods*, *1*, 477–491.

[CR36] James, W. (1950). The Principles of Psychology (Vol. 1). Dover Publications. (Original work published 1890).

[CR37] James, W. (2000). Humanism and truth. In G. Gunn (Ed.), *Pragmatism and other writings* (pp. 146–167). Penguin Books. (Original work published 1909).

[CR57] K Palmer, D. (2004). On the organism-environment distinction in psychology. *Behavior and Philosophy*, *32*(2), 317–347. JSTOR.

[CR38] Kosslyn, S. M. (1981). The medium and the message in mental imagery: A theory. *Psychological Review*, *88*(1), 46–66.

[CR39] Kosslyn, S. M., Ball, T. M., & Reiser, B. J. (1978). Visual images preserve metric spatial information: Evidence from studies of image scanning. *Journal of Experimental Psychology: Human Peception and Performance*, *4*(1), 47–60. 10.1037//0096-1523.4.1.47 627850

[CR40] Kvale, S. (1964). Husserls, heideggers Og marx’s Betydning for psykologien. *Nordisk Psykologi*, *16*, 186–198.

[CR41] Landecker, H. (2016). Antibiotic resistance and the biology of history. *Body & Society*, *22*(4), 19–52. 10.1177/1357034X14561341 28458609 10.1177/1357034X14561341 PMC5390938

[CR42] Latour, B. (1996). Cogito ergo sumus! A review of ed Hutchins cognition in the wild. *Mind Culture and Activity1*, *3*(1), 54–63.

[CR01] Lave, J. (1988). Cognition in practice: Mind, mathematics and culture in everyday life. Cambridge university Press.

[CR43] Lave, J. (2009). Situeret Læring Og Praksis i forandring. *Nordiske Udkast*, *37*(1). 10.7146/nu.v37i1.7255. Article 1.

[CR44] Lave, J. (2011). Apprenticeship in critical ethnographic practice (T. P. Gibson, Ed.). University of Chicago Press. https://press.uchicago.edu/ucp/books/book/chicago/A/bo8661187.html

[CR45] Lave, J., & Packer, M. (2008). Towards a social ontology of learning. In K. Nielsen, S. Brinkmann, C. Elmholdt, L. Tanggaard, P. Musaeus, & G. Kraft (Eds.), *A qualitative stance. Essays in honor of Steinar Kvale*. Aarhus University.

[CR46] Lave, J., & Wenger, E. (2003). Situeret læring—Og andre tekster. Hans Reitzels Forlag. (Original work published 1991).

[CR47] Leary, D. E. (2018). *The Routledge guidebook to james’s principles of psychology*. Routledge.

[CR48] Lefebvre, H. (1987). *The everyday and everydayness* (p. 73). Yale French Studies.

[CR49] Luria, A. R. (1976). *Cognitive development: Its cultural and social foundations*. Harvard University Press.

[CR50] McGann, M., Di Paolo, E. A., Heras-Escribano, M., & Chemero, A. (2020). Editorial: Enaction and ecological psychology: Convergences and complementarities. *Frontiers in Psychology*. 10.3389/fpsyg.2020.617898. 11https://www.frontiersin.org/articles/33324310 10.3389/fpsyg.2020.617898 PMC7725682

[CR51] Menary, R. (2007). Cognitive integration. *Palgrave Macmillan UK*. 10.1057/9780230592889

[CR52] Menary, R. (Ed.). (2010). *The extended Mind*. MIT Press.

[CR68] Møller, S. S. E., Bengtsen, & Munk, K. P. (Eds.). Metodefetichisme (pp. 103–121). Aarhus Universitetsforlag.

[CR53] Newen, A., Gallagher, S., & De Bruin, L. (2018). 4E cognition: Historical roots, key concepts, and central issues. In A. Newen, De L. Bruin, & S. Gallagher (Eds.), *The Oxford handbook of 4E cognition* (pp. 2–16). Oxford University Press. 10.1093/oxfordhb/9780198735410.013.1

[CR54] Nielsen, K. (1999). *Musical apprenticeship: Learning at the academy of music as socially situated*. Psykologisk Institut.

[CR55] Osbeck, L., & Nersessian, N. (2013). Situating distributed cognition. *Philosophical Psychology*, *27*(1), 82–97. 10.1080/09515089.2013.829384

[CR56] Packer, M. J., & Goicoechea, J. (2000). Sociocultural and constructivist theories of learning: Ontology, not just epistemology. *Educational Psychologist*, *35*(4), 227–241. 10.1207/S15326985EP3504_02

[CR58] Reed, E. S. (1986). Seeing through history. *Philosophy of the Social Sciences*, *16*(2), 239–247. 10.1177/004839318601600208

[CR59] Richards, G. (2009). Putting psychology in its place: Critical historical perspectives (3rd ed.). Routledge. 10.4324/9780203854600

[CR60] Roberts, T., Krueger, J., Glackin, S., & Philosophy (2019). *Psychiatry & Psychology*, 26(3), E–51.

[CR61] Rock, I., & Palmer, S. (1990). The legacy of gestalt psychology. *Scientific American*, *263*(6), 84–91. 2270461 10.1038/scientificamerican1290-84

[CR63] Schraube, E. (2013). First-person perspective and sociomaterial decentering: Studying technology from the standpoint of the subject. *Subjectivity: International Journal of Critical Psychology*, *6*(1), 12–32. 10.1057/sub.2012.28

[CR64] Sterelny, K. (2010). Minds: Extended or scaffolded? *Phenomenology and the Cognitive Sciences*, *9*(4), 465–481. 10.1007/s11097-010-9174-y

[CR67] Thisted, J. (2015). Metodespørgsmålet Og praksisfilosofien: Bidrag Til En Kreativ Metodeforståelse. In J. E.

[CR69] Thompson, E. (2007). *Mind in life*. Harvard University Press.

[CR72] Winther, I. W. (2017). Hverdagsliv—Trivialiserede praksisser. In E. Gulløv, G. B. Nielsen, & I. W. Winther (Eds.), Pædagogisk antropologi. Tilgange og begreber (pp. 27–39). Hans Reitzels Forlag.

